# Toward a framework of ambiguity: a qualitative understanding of healthcare policy design and governance mechanism in China

**DOI:** 10.3389/fpubh.2025.1666193

**Published:** 2025-10-21

**Authors:** Yuanbo Qi, Aijun Hu, Panpan Huang

**Affiliations:** College of Humanities, Donghua University, Shanghai, China

**Keywords:** public health policy, strategic ambiguity, behavioral response, health governance, health systems

## Abstract

**Introduction:**

This study explores the strategic and deliberate usage of ambiguity in Chinese public health policy as a governmental instrument.

**Methods:**

By analyzing 128 official public policy documents and 30 participant interviews as primary evidence, this study developed a three-tier coding instrument to capture the underlying categorizations, strategic functions, and behavioral responses.

**Results and discussion:**

The findings of this study indicate that ambiguity, categorized into five types—“*Elasticating*,” “*Generalizing*,” “*Overloading*,” “*Substituting*,” and “*Intensifying*”—with “*Elasticating*” being the most predominant, facilitated the diffusion of accountability, the shifting of responsibility, and the flexibility of interpretation. This research makes a significant contribution to the field of public health governance by redefining policy ambiguity as a complex, integrated mechanism of problem-solving that is rooted in the behavioral, institutional, and bureaucratic contexts of public health operations in China, rather than as a systematic failure.

## 1 Introduction

Policymaking environment, in many places over the globe, operates within a paradoxical space of *constrained plurality*—a governance condition in which a limited range of disagreement is tolerated, yet foundational critiques are systematically excluded from public deliberation (1–3). While the presence of regulated discursive openness has been documented in empirical contexts, the precise governance mechanisms that maintain this equilibrium of control and flexibility remain insufficiently explored. Among these mechanisms, strategic policy ambiguity stands out as both ubiquitous and underdeveloped. Often dismissed as an administrative flaw or the result of bureaucratic inefficiency, ambiguity in Chinese health policy may, in fact, function as a deliberate design feature—a flexible tool for managing complexity, sustaining symbolic authority, and enabling adaptive governance across implementation systems.

This study begins with the observation that the consequences of ambiguity might most visible be at the implementation level. Hospital staff, grassroots workers, and emergency responders frequently face unclear or conflicting instructions, resulting in interpretive labor, discretionary judgment, and emotional stress. While ambiguity in governance has been explored in Western policy settings, it remains undertheorized in the Chinese context—particularly in health administration, where policy signals are deeply embedded in evolving institutional hierarchies and expectations. As such, ambiguity not only influences *how* policies are delivered but also *who* is held accountable and *when* clarity is intentionally deferred.

Building on prior research that examined how signaling and discursive health policy affect China (4), this study transitions from a macro-level analysis of policy discourse to a micro-level investigation of policy ambiguity as a structural and behavioral phenomenon. Our goal is to uncover how ambiguity operates across both textual and performative layers of governance and to examine its role in shaping frontline administrative behavior. Rather than treating ambiguity as merely an absence of clarity, this paper understands it from a strategic form of governance that links institutional intent with behavioral outcomes. To pursue this aim, this study develops a three-tiered analytical framework that maps the relationships between the linguistic form of ambiguity, its strategic function in governance, and the behavioral response of policy implementers. The study combines qualitative content analysis of 128 government-issued health policy documents with interviews from 32 frontline administrators, including trainees, temporary staff, and healthcare facility workers. By building and applying a structured coding instrument, we are able to identify recurring patterns of ambiguity—categorized into five primary types and to understand how these patterns are received, translated, or resisted in practice.

### 1.1 Research questions

Ambiguity in Chinese governance is a deliberate and systemic strategy, yet its structural role in health policy design remains largely underexplored. A wide range of literature has documented the intentional use of ambiguity within China’s policy and governance systems. Ang (2) frames ambiguity and clarity as coexisting logics in Chinese legislative communication, treating them as adaptive tools in authoritarian statecraft. Zhan and Qin (5) similarly interpret policy ambiguity as a political discipline, used to retain control during the execution of complex mandates. Kostka and Hobbs (6, 7) offer further insight into this mechanism through studies on energy efficiency, demonstrating how local experimentation and “political guarantees” are used to navigate ambiguous directives. Liu, Tang, and Lo (8) extend this argument to the environmental sector, showing that ambiguity facilitates discretion in enforcement. Dai and Taube, focusing on linguistic nuance, reveal how semantic vagueness in policy documents enables flexible interpretation and implementation at the local level (9).

In the specific context of social services, Guo and Ba (10) use a conflict model to illustrate how ambiguity in pension policy is perceived and reinterpreted by street-level actors. Müller (11) highlights how village doctors navigate institutional uncertainty in China’s healthcare system, while Li et al. (12) show how ambiguity creates overlapping professional boundaries between pharmacists and other clinicians. Zhu, Li, and Pawson (13) further complicate the picture by documenting implementation dilemmas in housing policies. Meanwhile, Han (14) discusses how ambiguity affects individual decision-making in healthcare, though without connecting this to institutional design. Hu and Ji (15) focus on China’s international strategy, but their observations about symbolic ambiguity in external-facing policy discourse offer useful theoretical parallels. Horowitz (16) provides a comprehensive analysis of US strategic ambiguity regarding Asia Pacific geopolitical issue. Underscoring how ambiguity can serve both tactical and symbolic functions in high-stakes policy environments.

Despite these valuable contributions, two critical gaps remain. First, existing literature tends to focus on sector-specific, micro-level phenomena, without developing an integrated understanding of how ambiguity is structurally embedded across the entire governance architecture, especially in the health sector. Second, few studies link policy ambiguity to deliberate systemic design, overlooking how top-level decision-makers deploy ambiguity as a strategic governance tool in both policy formulation and operational implementation. This lacuna is particularly urgent given recent global scrutiny of China’s health governance (5, 17). To bridge these gaps, this study examines the intentional incorporation of ambiguity into Chinese health policy, analyzing its discursive construction and strategic functions as a governance mechanism. It moves beyond fragmented or surface-level observations to uncover how ambiguity is institutionalized and normalized in policy language, and how this shapes cognitive, operational, and ethical dimensions of healthcare delivery. This research addresses the following questions:

- RQ1: What are the primary categories and structural patterns of ambiguity that are prevalent in Chinese health policy documents?
- RQ2: What strategic functions do these forms of ambiguity serve in the policymaking and implementation process?
- RQ3: How do frontline implementers cognitively and behaviorally react to ambiguous policy directives?

By answering these questions, this research contributes to a more comprehensive theorization of ambiguity as a governance tool and offers empirical insights into how it shapes both the symbolic and operational dimensions of policy implementation. This research more broadly contributes to the comprehension of policy ambiguity, particularly in the context of China’s healthcare governance. It introduces a three-level analytical model that establishes a connection between the form of ambiguity (*what it is*), the strategic function (*why it is deployed*), and the behavioral response (*how it is received and adapted*). This model allows for a structured approach to the analysis of the political and administrative logic that underpins ambiguous or fluid policy language. The study empirically develops and implements a novel coding instrument that classifies five primary categories of policy ambiguity identified through content analysis of official health policy documents. The research contributes to more comprehensive debates regarding administrative adaptation, policy understanding, and bureaucratic control by shedding lights upon discovering how ambiguity maintains its political resilience at policy development.

## 2 Materials and methods

### 2.1 Theoretical framework

This study adopts an integrated approach grounded in Behavioral Public Policy (BPP) to examine how strategic ambiguity in Chinese public health policy is both designed and experienced. Drawing specifically on Gopalan and Pirog’s structured framework for applying behavioral insights (18), we distinguish three sequential phases of the policy process: ex-ante policy analysis, policy design, and ex-post policy review. This framework aligns with a broader shift in policy research that incorporates behavioral insights to recognize the cognitive limitations, emotional reactions, and decision-making environments of policy actors (19–21). Rather than viewing ambiguity as a failure of clarity, we conceptualize it as a policy variable that can be deliberately engineered or inadvertently produced—shaping how implementers interpret, enact, or resist policy signals. This theoretical model provides not only a structure for coding qualitative data but also a lens for interpreting the institutional and individual-level dynamics underpinning ambiguity (22).

Operationally, the three-part framework informs our coding and analysis. “Ambiguity formulation” maps onto ex-ante institutional and cognitive constraints such as role confusion, inconsistent mandates, or information gaps. “Strategic intent” captures midstream design-level choices where ambiguity is embedded for flexibility, risk diffusion, or symbolic messaging. “Behavioral responses” reflect how frontline implementers react—ranging from avoidance and compliance to active reinterpretation and contestation. All interview transcripts and policy documents were interpreted using this triadic structure, enabling the analysis to move fluidly between micro-level sensemaking and macro-level policy design. The framework also helps uncover how ambiguity is normalized and routinized through everyday practice, producing feedback loops that reinforce the very uncertainties frontline workers must navigate.

Finally, this framework supports a more nuanced understanding of how policy ambiguity contributes to symbolic governance and institutional legitimacy. Ambiguity is not only a cognitive or administrative challenge—it can serve as a symbolic resource that helps upper-level authorities maintain plausible deniability, flexible interpretation spaces, or emotional distance from contested decisions. Meanwhile, it imposes substantial emotional and ethical burdens on frontline actors who must interpret unclear directives in real-world, often high-stakes settings. By analyzing how ambiguity is staged, reproduced, and responded to across policy stages, the framework contributes to broader debates on governance opacity, hierarchical coordination, and implementation politics. Importantly, it also reveals how ambiguity can become self-reinforcing: the lack of clarity at the top encourages adaptive behaviors at the bottom, which in turn stabilize the ambiguous system through routinized improvisation. This recursive pattern—where structural vagueness and street-level interpretation feed into each other—helps explain the persistence of ambiguity in otherwise modernized and bureaucratically rational policy environments.

### 2.2 Research design

The primary dataset consists of National Chinese health policy documents issued between 2019 and 2024. Policy data from the National Health Commission is found on their official website (see *guifanxingwenjian* from https://www.nhc.gov.cn/wjw/gfxwjj/list.shtml, accessed on 6 June 2024). The process entails the search of this official website to locate the primary documents that denote their official policies updated on a regular basis. These updates are typically released on a monthly basis, with 8 to 10 formal documents being issued. The entire process was conducted in July 2024, and we employed random sampling to acquire Chinese health policy documents. We conducted a thorough examination of the extensive data that was gathered, and we continued the analysis until theoretical saturation was achieved. The data processing was subsequently conducted after the data collection was completed.

In addition to policy text analysis, we conducted interviews with 32 (*N* = 32) individuals directly involved with the interpretation of health policy in hospitals and administrative settings. The interview participants were purposively selected to capture a broad spectrum of perspectives from individuals directly involved in the implementation of health-related policy directives. The sample included: (1) university student internship temporarily assigned to administrative roles within public health contexts; (2) university faculty members engaged in institutional health governance; (3) hospital volunteers and contracted support personnel; (4) licensed medical practitioners; and (5) district-level public health office staff responsible for frontline policy execution. The research conducted snowball sampling in metropolitan Shanghai. Interviewees remained anonymous, each assigned a unique identification number (Table 1).

**Table 1:** Participant demographics.

Characteristic	N (%)
Nationality	
Chinese	26 (81)
Pakistani	3 (9)
African	3 (9)
Education level	
Undergraduate students	16 (50)
Postgraduate students	16 (50)
Age (years): median (Q1–Q3)	32 (21–35)
Gender	
Female	26 (81)
Male	6 (19)
Policy encounter	
Reported experiencing ambiguous policy	31 (97)
No direct experience (assumed scenario)	1 (3)
Primary location of policy encounter	
Mainland China	30 (94)
Abroad or hypothetical context	2 (6)

### 2.3 Coding and analytical procedure

This study adopts a qualitative interpretivist design using a three-tiered coding instrument to examine policy ambiguity in Chinese health governance. Data were drawn from two sources: central-level policy documents and interview transcripts with frontline health-sector actors. This dual-source design allowed top-down and bottom-up triangulation: ambiguity types were first derived from national texts and then verified against their appearance in local practice. The aim was not only to catalog discursive ambiguity but to test whether textual features were transmitted intact through the governance chain and to document the issues they generated for implementers. By linking document analysis with field narratives, the procedure shows how central framing structures street-level discretion. The three tiers—ambiguity types, strategic functions, and behavioral responses—map the pathway from production to enactment, enabling integrated verification rather than treating ambiguity as rhetorical artifact.

Tier One inductively identified five ambiguity types in central policy texts: directive, temporal, interpretive, procedural, and evaluative. These were derived through close reading attentive to semantic patterns, repetition, vagueness, and framing shifts. Treating documents as governance blueprints, the codes reflected how authorities balanced symbolic reassurance with operational flexibility. Tier one thus served as a benchmark for verification, each type functioning as a hypothesized “carrier” expected to travel downward. A preliminary list mirrored central framing. This baseline enabled examination of whether frontline accounts reproduced, reinterpreted, or resisted the same ambiguity signatures, transforming a descriptive taxonomy into a testable reference model.

Tier Two analyzed the strategic functions of ambiguity, inferred from central texts and aligned with implementers’ accounts. Three functions dominated: political buffering, symbolic reassurance, and flexibility for implementation. Field evidence confirmed that these functions were legible and actionable. Political buffering appeared as diffuse accountability; symbolic reassurance as steady messaging amid uncertainty; and flexibility as permissible discretion under resource limits. This convergence verified that strategic intent embedded in central language permeated everyday decision-making, confirming that design and practice were coupled and that central ambiguities were indeed delivered downward.

Tier Three traced recurring behavioral responses—evasion, routinization, and deferral—through which implementers navigated ambiguity. These responses confirmed that document-derived ambiguities were operative in practice, though refracted by local constraints, workload pressures, and risk perceptions. They also revealed practical problems, such as uncertainty over priorities, sequencing, and compliance thresholds. By documenting repeated coping patterns, Tier Three confirmed continuity between textual production and enactment, showing ambiguity’s systemic effects on problem-solving and discretion.

Coding followed an iterative procedure in NVivo 14 to secure saturation and theoretical consistency. A preliminary list derived from documents was refined through two further rounds applied to interviews, adapting categories where narratives warranted revision while preserving typological alignment (23). Saturation was judged by stability of categories and recurrence across cases; theoretical consistency by coherence between types, functions, and behaviors. Reliability was secured through independent coder review; discrepancies were resolved by discussion, with inter-coder reliability reaching Cohen’s Kappa 0.80 (24). A pilot application preceded the full study, prompting two structural revisions to sharpen tier distinctions and clarify coding rules (25). The validated instrument is presented in Table 2. Scope was intentionally limited: mid-level administrative actors and stakeholder models were excluded. Ambiguity was examined from central texts to local implementation, but intermediary communication layers remain outside the design. This leaves open questions for future studies that may aim to trace how ambiguity is co-produced and diffused across the full policy cycle, mediating both governance intent and implementer challenges.

**Table 2:** Coding instrument of ambiguity types in Chinese health policy (*N* = 128).

Main code (CAPS)	Sub-code	Definition	Trigger criteria
Elasticating	Broad Task Framing	Use of catch-all terms to capture undefined, multitasking roles	Vague phrases like “daily operation,” “assist with management” used without specification
	Policy Scope Stretching	Extending a policy’s applicability far beyond its initial or stated scope	When vague mandates are applied across unrelated tasks or settings
Generalizing	Moral Abstraction	Evoking collective values or ideologies to suppress questioning of vague content	Use of slogans like “health for all,” “for social stability” in place of actionable items
	Principle Without Protocol	Affirming values without outlining procedures or accountability mechanisms	Idealistic language without stepwise implementation or monitoring
Intensifying	Information Saturation	Releasing dense updates and complex notices in rapid succession	Multiple announcements with unclear or overlapping deadlines
	Time-Pressured Decisions	Pressuring implementers to act before clarification is possible	Instructions with urgent or emergency framing but unclear content
Overloading	Multi-Objective Fusion	Combining unrelated policy goals in a single document or directive	A single task list includes performance, ideology, risk, admin duties
	Complex Policy Stacking	Layering rules and tasks across multiple notices without hierarchy	Successive documents reference each other without summary or integration
Substituting	Terminological Shift	Replacing precise terms with more elastic or less accountable ones	Clear mandates become “recommendations,” “support,” or “advice”
	Euphemistic Reframing	Using softer or more neutral language to mask control or coercion	Directive language softened through friendliness or voluntarism

### 2.4 Institutional review board statement

This study was granted an exemption in accordance with the “Measures for Ethical Review of Life Science and Medical Research Involving Humans” (Article 32, Chapter 3) issued jointly by the Chinese Health Commission, Ministry of Education, Ministry of Science and Technology, and the Bureau of Traditional Chinese Medicine ([2023] No. 4, see https://www.gov.cn/zhengce/zhengceku/2023-02/28/content_5743658.htm, accessed on 2 June 2024). Specifically, this study qualifies for exemption condition (2), which applies to research conducted “using anonymized information data” (p. 13). This research presents no more than minimal risk. All direct identifiers (such as names, ID numbers, contact information) and indirect identifiers were removed, ensuring the data is fully anonymized and cannot be re-identified. This study was conducted in full accordance with the Declaration of Helsinki. All methods were carried out in accordance with relevant guidelines and regulations. The informed consent was obtained.

## 3 Results

### 3.1 Ambiguity types and frequencies

Table 3 summarizes the distribution of five primary types of policy ambiguity identified across 128 coded instances in Chinese health policy texts. *Elasticating* Ambiguity emerged as the most frequent category, accounting for 37 instances (29%). *Generalizing* Ambiguity accounted for 28 instances (22%) and reflected an intentional use of normative or ideological language — particularly through *Moral Abstraction* (13%) and *Principle Without Protocol* (9%). *Intensifying Ambiguity* (23%) was frequently triggered by time pressure and information overload, as evidenced by *Information Saturation* (12%) and *Time-Pressured Decisions* (11%). *Overloading* (16%) and *Substituting* Ambiguity (10%) were also visible.

**Table 3:** Types of policy ambiguity identified in Chinese health policy (*N* = 128).

Code	Sub-code	N (%)	Sum (%)
Elasticating	Broad Task Framing	23 (18)	37 (29)
	Policy Scope Stretching	14 (11)	
Generalizing	Moral Abstraction	16 (13)	28 (22)
	Principle Without Protocol	12 (9)	
Intensifying	Information Saturation	15 (12)	29 (23)
	Time-Pressured Decisions	14 (11)	
Overloading	Multi-Objective Fusion	12 (9)	21 (16)
	Complex Policy Stacking	9 (7)	
Substituting	Terminological Shift	8 (6)	13 (10)
	Euphemistic Reframing	5 (4)	

### 3.2 Strategic functions of ambiguity

Table 3 outlines the strategic intents behind the deployment of different types of ambiguity in Chinese health policy. *Elasticating* ambiguity—represented here by the strategy of *Delegating*—was used to shift responsibilities downward. By framing roles with vague terms like “daily operations,” policymakers transferred interpretive labor and decision-making burden to implementers. *Generalizing* ambiguity served a *Justifying* purpose. By embedding policies in moral or ideological rhetoric, it discouraged questioning and normalized excessive demands. *Intensifying* ambiguity followed a strategy of *Overwhelming*. The cognitive excess that resulted from the frequent sense of urgency updates and inconsistent directives left workers with little time for reflection. In contrast, *Overloading* ambiguity relied on *Obfuscating*, combining unrelated goals to diffuse accountability and deflect blame. Those compound directives allowed supervisors to reinterpret misconduct as misunderstanding. Finally, *substituting* ambiguity employed a *Reframing* strategy. It increased compliance by making coercive measures appear gentle by softening mandates, such as reframing “quarantine” as “health support.” Table 4 emphasizes the manner in which ambiguity is designed to function as a form of soft power, utilizing language to transfer control, conceal demands, and compel silent consent.

**Table 4:** Strategic purposes of ambiguity.

Strategy code	Manifestation	Illustrative quote
Delegating	Broad and vague policy terms (e.g., “daily operation”) transfer interpretation to lower-level actors, pushing down responsibility and increasing task ambiguity.	“When they recruited me for daily operations I thought I would only translate. The staff then assigned me duties that included both cleaning tasks and reception work as well as English teaching responsibilities. I asked for clarity but they only repeated the phrase “support the office.”—ID 1
Justifying	Moral-ideological framing (e.g., “for harmony,” “health for all”) embeds policies in ethical discourse to discourage resistance and make abstract compliance seem virtuous.	“They explained the necessity for universal contribution to build a balanced environment. They presented my doubts about the workload as an act of selfishness. The discussion shifted from workload concerns to focus on core values and organizational loyalty. The term ‘social harmony’functions as a shield to hide insufficient planning although it remains unchallengeable.”—ID 6
Overwhelming	Rapid policy updates and urgent deadlines create cognitive overload, forcing actors to comply without full processing.	“They gave us three notices within 24 h, all with different requirements. Each one said ‘immediate action required’ and none of them matched. I did not even know which one to follow. I just picked one and hoped for the best, because there was no time to ask anyone.”—ID 19
Obfuscating	Combining multiple goals or rules in one directive fragments responsibility, enabling blame shifting and interpretive escape routes.	“Student management along with data security and promotional tasks were presented in a single document with no clear distinction between them. The supervisor told me I had failed to comprehend the ‘bigger picture’ after a mistake occurred. The picture remained unexplained to anyone who asked. The same paragraph produced different interpretations among all workers.”—ID 12
Reframing	Terminological shifts turn hard mandates into softer-sounding suggestions (e.g., “quarantine” → “health support”), increasing policy acceptability.	“At first they imposed a mandatory daily check-in program. The term changed from ‘health encouragement message’ to ‘health encouragement message’ without any change in the expected workload. The name changed, but the pressure stayed.”—ID 21

### 3.3 Behavioral response to ambiguity

The third significant result pertains to the behavioral and cognitive responses of policy implementers to ambiguous directives. Five distinct behavioral patterns were identified, as illustrated in Table 5. The proactive strategy of *clarifying* involved implementers seeking explanations to make sense of ambiguous language in order to prevent errors and mitigate future accountability risks. *Complying* was a passive but pragmatic response, while *avoiding* indicated disengagement, as implementers withdrew from tasks that felt ethically or cognitively untenable. *Ignoring* was indicative of a deliberate filtering process, in which employees de-prioritized tasks that were perceived as non-essential or unmonitored, thereby enabling them to conserve cognitive energy for tasks with more explicit enforcement. Lastly, *confronting* necessitated moral resistance, as participants directly confronted ambiguity when it endangered their dignity or equity. Collectively, these behaviors established a continuum from passive endurance to active resistance, demonstrating the influence of cognitive burden, perceived risk, and institutional pre-existing culture on the behaviors in response to this ambiguity.

**Table 5:** Behavioral responses to ambiguity.

Behavior	Cognitive mechanism	Illustrative quote
Clarifying	Sense-making under uncertainty	“The initial meaning of ‘assist with communications’ was unclear to me so I requested my manager to explain it three times. The different answers she provided created more uncertainty for me. I kept asking questions because I wanted to prevent mistakes which would result in future blame.”—ID 3
Complying	Heuristic acceptance and risk aversion	“My approach to unclear instructions such as ‘be proactive’ and ‘wait for approval’ is to perform both actions. My goal is to maintain employment while avoiding any difficulties at work. The process remains my best choice although it drains my energy.”—ID 6
Avoiding	Emotional withdrawal from cognitive dissonance	“I did not confront anyone. I just slowly reduced my involvement. I explained that I was preoccupied with school responsibilities before making my departure. Emotionally, I had already exited long before.”—ID 2
Ignoring	Strategic de-prioritization	“When ambiguity reaches an extreme point it loses all meaningful value. I completely ignore the part which states “proactive engagement maintenance” because it seems meaningless to me. The instruction demands me to act both independently and following a script.”—ID 19
Confronting	Moral dissonance and self-assertion	“After they tried to present everything as learning opportunities I confronted them directly. I named it exploitation because it is unpaid work with no definition. I voiced my words in front of everyone which helped me regain my dignity slightly.”—ID 16

**Figure 1:**
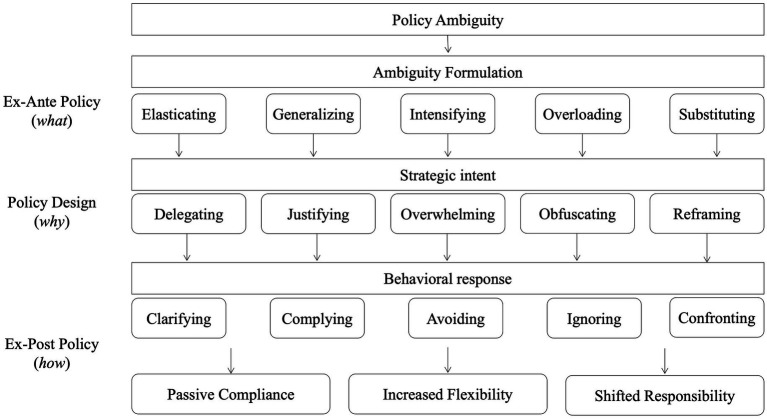
A conceptual mechanism of ambiguity formulation, strategic intent, and behavioral response in policy implementation.

Figure 1 revealed a cyclical mechanism of reflective adaptation that connects the form, function, and effect of ambiguity in Chinese health policy implementation. This figure outlines three interconnected layers: (1) End-Policy Ambiguity, representing the five identified ambiguity types embedded in central policy texts and being verified by the frontline participants during interview of this study; (2) Strategic Policy Design, where the intentional deployment of these ambiguities serves administrative goals; and (3) Ex-Post Behavioral Response, which reflects how frontline implementers interpret and navigate these ambiguities in daily practice. These ambiguity types reflect deliberate strategic rationales: delegating responsibility, justifying mandates, overwhelming implementers, diffusing accountability, and reframing coercion. Together, they form an interlinked package for flexible policy control. When implementers encounter these forms, they exhibit one of five behavioral-cognitive responses: *clarifying*, *complying*, *avoiding*, *ignoring*, or *confronting*. These responses reflect adaptive psychological mechanisms of sense-making, risk aversion, or moral resistance. The cumulative effect of this cycle results in three significant institutional outcomes: passive procedural compliance despite confusion, increased local flexibility in interpretation and execution, and upward responsibility deflection, where lower-level actors absorb blame while shielding policymakers from fallout. This loop subsequently reinforces itself, as perceived implementer adaptability and ambiguity tolerance legitimize additional ambiguous policymaking. While this visual model does not substitute for cross-tabulated analysis or interactional mapping, it helps clarify how policy ambiguity flows through the governance pipeline and provides a conceptual platform for future analytical expansion.

## 4 Discussion

### 4.1 Interplay between policy ambiguity and behavioral responses

This study acknowledges that the five identified types of policy ambiguity—*elasticating, generalizing, intensifying, overloading,* and *substituting*—rarely appear in isolation during real-world governance processes. Instead, they often operate in layered, intertwined forms, compounding the interpretive burdens experienced by frontline implementers. For instance, *elasticating* often stretches task definitions under broad terms such as “daily operations,” while *generalizing* anchors those tasks in moral or cultural values. *Intensifying* and *overloading* may simultaneously create a highly pressurized working environment through compressed timelines and excessive duties. *Substituting* further exacerbates the situation by reframing professional obligations as private favors. These ambiguity types not only overlap in function but also reinforce each other, leading to compounded confusion, blurred role boundaries, and emotional exhaustion among implementers. Recognizing such interactivity is vital for accurately capturing the operational realities of health governance in China.

A vivid case exemplifying these intersecting ambiguities comes from Participant ID7, a new hire working as a translator in a street-level healthcare office in Shanghai. Initially promised translation duties, this participant was warmly welcomed with personal gestures such as snacks and dinner —subtly introducing *substituting* by transforming formal workplace roles into private relationships. Her job soon expanded without formal notification: beyond translation, the participant was asked to perform various secretarial and logistical tasks, justified through elasticating terms like “assisting daily operations.” These undefined phrases were later stretched to cover almost all departmental tasks. *Overloading* followed, as the participant received an unmanageable quantity of work with tight deadlines—often being asked to translate 20 slides within hours. When the participant raised concerns, the leadership deployed *generalizing* discourses, invoking traditional values like “selfless dedication” (*fengxian*) and collective responsibility (*qingfen*). This was combined with *intensifying* type, framing her grievances as a failure to “keep up” under time pressure. Feeling overwhelmed by the combination of these ambiguities, ID7 ultimately resigned, stating: “This is not what I signed up for.” Her experience typifies the psychological and practical toll of compound ambiguity.

In addition, this study identifies five common behavioral responses to ambiguous policy environments: *clarifying, complying, ignoring, avoiding,* and *confronting*. Among these, *clarifying* emerges as a universal step when ambiguity is encountered, applicable across all five types. *Complying* is often the default choice under pressure, though many participants demonstrate avoiding behavior when they believe tasks exceed their mandate. *Ignoring* might especially be common in the face of *overloading* or *intensifying*, where participants disengage silently from unrealistic expectations. *Confronting*—while rare—is most emotionally and professionally risky, particularly when ambiguity stems from substituted private relationships, where confrontation may jeopardize informal harmony. Nevertheless, certain participants—such as ID7—explicitly drew boundaries in response to ambiguous directives, indicating that ambiguity alone does not rigidly determine behavioral responses. While all five ambiguity types are capable of triggering a wide range of reactions, the frequency and intensity of each response may vary. What ambiguity does shape, however, is the emotional labor and strategic calculus underpinning these actions: the decision to comply, resist, or disengage may often be less about the ambiguity type itself and more about how individuals perceive its implications for personal risk, institutional norms, and moral obligation.

While this study offers qualitative insight into the interactions between ambiguity types and their behavioral outcomes, a more systematic investigation is needed to map these relationships precisely. Future research may adopt quantitative or mixed-method approaches—including cross-tabulation, regression modeling, or comparative analysis—to assess whether certain ambiguity types consistently lead to specific behavioral responses. For instance, is *generalizing* more likely to elicit *compliance* due to its moral undertone? Does *overloading* result in *ignoring* because of task fatigue? Answering such questions would require a shift of research questions and a redesigned interview protocol, targeting interaction effects and response prevalence. Nevertheless, the current study lays the conceptual groundwork by identifying both the typologies of ambiguity and the universe of plausible reactions, opening the path for future comparative and statistical inquiry into policy ambiguity as a behavioral governance mechanism.

### 4.2 Ambiguity as symbolic control

Health policy ambiguity is not accidental but strategically crafted to maintain symbolic control while evading rigid accountability. First, it challenges conventional policy analysis frameworks that treat clarity as a normative ideal. Instead, ambiguity becomes a tool of elastic governance that enables political and institutional actors to maintain symbolic control while avoiding rigid commitments. This repositions policy as a mechanism of authority preservation.

Each type of ambiguity identified is linked to a rhetorical or operational strategy, such as delegating responsibility or reframing authority. Policymakers employ ambiguity as a strategy to maintain symbolic control and circumvent rigorous commitments, rather than pursuing clarity. The foundational concept and ambiguity in public policy facilitate political maneuvering and adaptive choice in governance (26). The strategies pertain to the implementation and governance of policies, with a particular emphasis on the strategic responses of those responsible for executing and implementing policies, including delegated authorities and agent agencies, in conjunction with policy inconsistency (27). This study reinforces those findings by demonstrating how delegated agents utilize ambiguity to diffuse responsibility and manage inconsistent policies. Ambiguity in policy and agency-based experiences may serve as solutions to governance and government operations challenges, presenting it as a rhetorical issue rather than an experience that demands improvising (28). This study corroborates the assertion that ambiguity is not merely a defect or malfeasance, but rather a rhetorical device that is ingrained in governance practices (28). In neoliberal cities, ambiguity is agency through a critical optimist lens, and a device to define certain concepts and practices when associated with policy (29). This research contributes to this optimistic interpretation of ambiguity, which is particularly pertinent to urban or state systems that prioritize flexibility over prescription.

### 4.3 Ambiguity as risk displacement

Second, ambiguity enables risk transfer to implementers, empowering discretion but diluting accountability. This redistribution of responsibility downward allows upper-level policymakers to avoid direct accountability, enabling blame diffusion since it creates an implementation environment where execution is flexible, but responsibility is not reciprocal (30, 31). This study provides empirical support for those who attempt to understand how frontline actors use interpretive discretion under uncertainty (32). It adds a healthcare-specific example, showing how insurance staff must often reinterpret vague directives in real time. They take on the risk themselves without questioning it. Sometimes, when responsibility cannot be clearly assigned, the person who provided the policy interpretation aims to remain ambiguous as well—otherwise, they would be held responsible.

Though a limited number of clarifications, most people choose to comply with what they are requested. Even when policies and principles are not clearly defined, they still accept and execute them. Their cognitive process is on acceptance and risk aversion—they prefer stability over friction. *Avoidance* occurs when people have already attempted both compliance and clarification but doomed unsustainable. They usually respond through withdrawal or disengagement, functioning as a psychological or logistical escape from untenable tasks. *Ignoring* occurs when implementers have the choice to deprioritize certain tasks. This typically happens when they weigh their options and assess the risks involved. It is usually a calculated decision, where ambiguous tasks are seen as non-essential or low priority. *Confrontation* is not usually the first response when encountering ambiguous policy that lacks clear clarification. It occurs when ambiguity has led to exploitation, compromises, and unsustainable demands that one cannot choose to ignore or avoid. Certain implementers justify their confrontational stance on moral grounds, believing they have an ethical obligation to speak up.

- *“I always check before acting. I do not want to ‘guess’ and get punished. If the instructions are unclear, that is their problem, not mine.” (ID14) “Even when it makes no sense, I go along. Arguing is not worth it. I just follow and figure it out later.” (ID11) “The policy had five vague points. I had no idea what my task really was, but I filled out the form anyway—because that is what they expected.” (ID8).*

### 4.4 Ambiguity as institutional normalization

Third, and perhaps most critically, repeated exposure to ambiguity fosters routinized, normalized behaviors that institutionalize it. Implementers learn that ambiguity is to be endured, interpreted, or evaded—but rarely challenged. This leads to routinized behaviors such as silent compliance, informal clarification, or strategic avoidance. Over time, such patterns institutionalize ambiguity as part of the policy experience, reducing demands for reform or transparency.

The theoretical groundwork for understanding how ambiguity is embedded in institutions and becomes an expected element of governance. Connolly, for instance, argues that ambiguity is not an aberration but must be institutionally expressed (33). This is clearly demonstrated in the institutional normalization phenomenon and interconnections—a particularly permanent case in China. This directly supports this study’s claim that ambiguity becomes part of the policy experience, rather than an exception. Institutional power and the discourse of normalcy in European Union governance highlight how ambiguity is fueled and perpetuated by power structures that become normalized (34). These circular discourses are not passive cultural frameworks—their exclusive focus on logistical interpretations helps analyze structural factors that shape power dynamics. A discursive reinforcement is the process highly relevant to Chinese administrative narratives.

Regarding how daily routines, norms, and adaptive behaviors consolidate ambiguity within organizational life. Culture and ambiguity in knowledge-intensive firms by evaluating ambiguity routines in operations, organizational routines, and cultural effects (35). Culture reproduces itself based on its strength and acceptance, particularly regarding the promotion of loyalty, commitment, effectiveness, and stability. It impresses a familiarity and constancy where gender concerns were absent—a form of normative control whereby consultants operate freely and sometimes participate in regulating their own autonomy. This is relevant to how Chinese interns comply without challenging (36).

Studies that might not focus on ambiguity directly show how deviant practices, when routinized, normalize systemic incoherence. The normalization of corruption in organizations, and while not directly focused on ambiguity, they point out that when corruption or decisions produce positive outcomes, it tends to reinforce inherent ambiguity (37). There are certain correlations where deviant practices or subcultures arise to normalize ambiguity within institutions, similar to what we observe in the Chinese context. Occasionally, corruption might become normalized when it produces functional outcomes in certain political context (37). This study has shown a parallel picture: ambiguity can be normalized if it helps maintain institutional harmony or stability—even at ethical or operational costs.

Overall, the discussion has established three interlinked functions of policy ambiguity in China’s health governance. First, ambiguity is strategically employed to reinforce symbolic control and maintain the perceived legitimacy of governing institutions. Second, it operates as a mechanism of risk displacement, transferring the burden of interpretation and execution to street-level bureaucrats, thereby diffusing institutional accountability. Third, ambiguity becomes normalized through repeated administrative use, gradually forming an institutional habitus that embeds uncertainty into daily governance routines.

Beyond these empirical dimensions, however, ambiguity also entails significant normative consequences. Persistent vagueness in policy directives undermines procedural clarity, exposing frontline implementers to unclear mandates with limited recourse or justification. For citizens seeking direct communication, these ambiguities are often experienced as obfuscation or deflection—responses that resemble automated, impersonal exchanges (2, 38). Such dynamics may result in inequitable outcomes, where those lacking organizational protection face disproportionate burdens. Over time, these processes risk eroding public trust, particularly in crisis-driven or high-stakes policy contexts. While this study has focused primarily on the cognitive and operational aspects of policy ambiguity, future research should more explicitly explore its ethical implications, including how it affects transparency, perceived fairness, and institutional legitimacy in governance systems (39–41). These questions are central to a more nuanced understanding of how ambiguous policy instruments shape not merely implementation but also the ethical fabric of state-society relations.

## 5 Conclusion

This study sheds light on the structured yet covert role of ambiguity in Chinese health policy implementation. Through a content analysis of 128 policy texts and interpretive coding grounded in a theoretically developed framework, the research identifies five distinct types of *ambiguity—Elasticating, Generalizing, Intensifying, Overloading,* and *Substituting*—serving a strategic function, such as delegating responsibility, justifying mandates, or reframing coercion. In turn, frontline implementers exhibit five behavioral responses ranging from passive compliance to active resistance, including *clarifying*, *complying*, *avoiding*, *ignoring*, and *confronting*. These interactions form a cyclical mechanism whereby ambiguity enables flexible control while simultaneously displacing blame and inducing silent consent. In doing so, this study demonstrates that ambiguity in Chinese health policy is not an administrative flaw but a deliberate governance strategy. First, ambiguity operates as a symbolic tool of control, enabling political actors to maintain authority while avoiding rigid commitments. Second, it facilitates the downward transfer of risk and responsibility, leaving frontline implementers to navigate uncertainty and shoulder blame. Third, the repeated exposure to such ambiguity leads to behavioral normalization, embedding ambiguity into the institutional fabric of governance. Together, these mechanisms reveal ambiguity as a productive and routinized mode of governance in China’s health policy landscape—one that sustains flexibility, evades accountability, and discourages structural reform.

This study has several limitations that constrain its scope and invite further inquiry. First, the research is geographically and sectorally bounded drawing exclusively from urban and semi-urban health governance contexts in China. Such a narrow empirical focus may overlook the institutional variations and communicative dynamics present in rural areas, where administrative constraints and sociocultural factors differ markedly. Future research could extend this framework to other policy sectors—such as education, labor, or environmental governance—to assess its adaptability and broader relevance. Second, while this study traces how ambiguity is enacted at the frontline, it does not explore the meso-level mediation of ambiguity by middle-tier administrators. Future studies could examine how policy vagueness is reshaped or resisted at these intermediary layers of governance, which likely play a crucial role in implementation outcomes.

Moreover, this research emphasizes street-level dynamics but does not incorporate multi-actor governance frameworks. Future work may benefit from integrating models such as the Pentahelix approach, which emphasizes collaboration among government, civil society, academia, media, and business stakeholder types. Such an orientation would enable researchers to analyze how ambiguity is co-produced or challenged across policy cycle stages, including problem definition, design, and evaluation. Incorporating participatory or mixed-method approaches could also help investigate whether insufficient stakeholder engagement exacerbates policy vagueness or fosters downstream confusion. Additionally, although the study offers a robust typology of policy ambiguity, it does not explicitly gauge or measure how different types of ambiguity interact or influence implementer behavior. Future studies could explore hybrid ambiguity patterns—such as the co-occurrence of different types of ambiguity—and examine their compound effects on cognition and compliance. Finally, while this study briefly notes the ethical risks of persistent ambiguity, such as obscured transparency and eroded public trust, it does not systematically assess these normative implications. Further research should explore how ambiguity influences perceptions of fairness, legitimacy, and institutional accountability—especially in crisis or emergency governance contexts, where such concerns are likely to be most acutely exposed. Together, these future directions would significantly deepen our understanding of policy ambiguity across actors, institutions, and levels of governance.

However, despite these limitations, this study makes a significant theoretical and empirical contribution by bridging a critical gap in our understanding of how ambiguity operates at the intersection of policy design and frontline implementation. This study offers a grounded, empirically validated typology of ambiguity that is directly drawn from and tested against the lived experiences of frontline implementers. It demonstrates that ambiguity may not be a design flaw or a rhetorical tool but a dynamic and multifaceted governance mechanism that influences emotional labor, decision-making, and institutional navigation at the street level. By mapping five distinct types of ambiguity—*Elasticating, Generalizing, Intensifying, Overloading,* and *Substituting*—and exploring those possible behavioral consequences, this research provides a granular, actionable framework for understanding how vague or contradictory policy language is operationalized in existing political settings. It also contributes methodologically by integrating both policy texts and practitioner narratives, offering a richer view of the discourse–response nexus. In doing so, the study advances the field’s capacity to conceptualize ambiguity not only as a discursive construct but also as a site of contested governance and negotiated meaning within bureaucratic systems. Ultimately, this research contributes to a developing corpus of literature that may challenge the premise that clarity continually represents the policy ideal from a managerial viewpoint. In the Chinese context, ambiguity is not only functional but also structurally entrenched in the practice of control, adaptation, and symbolic governance. This research provides a conceptual and empirical foundation for reevaluating the ways in which policies are made, interpreted, and lived under Chinese administration by demonstrating how ambiguity operates as a strategic instrument and behavioral.

## Data Availability

The original contributions presented in the study are included in the article/supplementary material, further inquiries can be directed to the corresponding author.
